# Impacts of Segond Fractures on Anterior Cruciate Ligament Reconstruction Outcomes

**DOI:** 10.7759/cureus.56542

**Published:** 2024-03-20

**Authors:** Tetsuhiro Hagino, Satoshi Ochiai, Tetsuo Hagino, Naoto Furuya, Masanori Wako, Hirotaka Haro

**Affiliations:** 1 Department of Orthopedic Surgery, National Hospital Organization (NHO) Kofu National Hospital, Kofu, JPN; 2 Department of Orthopedic Surgery, University of Yamanashi, Chuo, JPN

**Keywords:** anterior cruciate ligament (acl) restoration, anterolateral structure, anterior cruciate ligament injury, knee joints, segond fracture

## Abstract

Introduction: Segond fractures, characterized by avulsion injuries at the lateral tibial condyle's anterolateral structure (ALS) attachment, often coincide with anterior cruciate ligament (ACL) injuries, potentially leading to knee instability. However, the influence of Segond fractures on knee stability after ACL reconstruction remains uncertain. Despite documented ALS reconstructions, there is a lack of consensus regarding the assessment of ALS failure and the criteria for surgical interventions. This study aimed to determine if Segond fracture presence impacts ACL reconstruction outcomes, utilizing patient-reported subjective assessments and healthcare providers' objective evaluations.

Materials and methods: This retrospective study encompassed 639 patients (328 males, 311 females; mean age 26.9 years) who underwent ACL reconstruction, with a follow-up of at least one year. Subjects were divided into two groups: Segond fractures diagnosed through radiographic findings (Group S+, n = 17) and no Segond fractures (Group S-, n = 622). Clinical evaluation included the 36-item Short Form Survey (SF-36), Lysholm score, visual analog scale (VAS) for knee pain, knee injury and osteoarthritis outcome score (KOOS), and knee instability assessment via Telos SE (Telos Japan, Tokyo, Japan). Statistical comparisons were performed between the two groups.

Results: At the final follow-up, all SF-36 subscales improved in all eight subscales compared to before surgery, reaching national standard scores; no significant inter-group differences were evident. Lysholm scores were 93.0 ± 12.1 (Group S+) and 91.7 ± 10.9 (Group S-) (P = 0.62), VAS for knee pain was 10.0 ± 18.0 (Group S+) and 11.9 ± 16.9 (Group S-) (P = 0.62), total KOOS was 89.0 ± 17.4 (Group S+) and 90.7 ± 9.9 (Group S-) (P = 0.39), and anterior tibial translation differences were 2.8 ± 3.0 mm (Group S+) and 2.7 ± 2.9 mm (Group S-) (P = 0.73). All these values represent postoperative measurements. No significant discrepancies existed between groups across evaluation methods.

Conclusions: This study's results suggest that Segond fractures have minimal impact on clinical ACL reconstruction outcomes, as assessed through both patient-reported subjective evaluations and objective healthcare provider evaluations. Segond fractures' significance in postoperative outcomes questions the necessity of ALS reconstruction.

## Introduction

In 1879, Paul Segond reported that strong internal rotation of the knee joint was associated with an avulsion fracture in the anterolateral aspect of the proximal tibia and that a shiny fascicle was attached to this site [1]. Subsequently, avulsion fractures from the lateral tibial condyle at the attachment of the anterolateral structure (ALS) commonly occurring with anterior cruciate ligament (ACL) ruptures are called Segond fractures, and their involvement in rotatory knee instability has been reported [2,3]. The ALS is composed of the superficial layer of the iliotibial ligament (ITB), the Kaplan fiber that attaches from the deep layer of the ITB to the posterior distal femur, the deep capsulo-osseous layer, and the anterolateral ligament (ALL) [4]. Kumahara et al. reported that the ALL regulates the internal rotation of the tibia and influences the pivot shift in knees with ACL injuries [5]. In addition, a detailed comparison of the ALL footprint and the site of Segond fracture by CT and MRI examinations of cadaveric knees showed that the site of Segond fracture concurred with the ALL footprint on the proximal tibia, proving that Segond fracture was a bony avulsion of the ALL [6].

However, whether the presence of a Segond fracture affects knee joint stability after ACL reconstruction remains unclear. Regarding this issue, ALS reconstruction has been utilized, but there is no consensus on the evaluation method for ALS failure or the indications for ALS surgical reconstruction.

In a previous study, we evaluated patients with ACL injury using the Medical Outcome Study 36-item Short Form Survey (SF-36), which is a patient-based questionnaire that evaluates QOL in fine detail and reports the importance of patients’ subjective evaluation in addition to doctor-based objective assessments [7-9]. However, there is no report on subjective evaluation using a patient-based assessment method after ACL reconstruction in patients with concomitant Segond fractures. The objective of this study was to investigate whether the presence of Segond fractures influences the outcomes of ACL reconstruction by analyzing the postoperative outcomes of ACL injuries evaluated subjectively by patients and objectively by health providers.

## Materials and methods

**Figure 1:**
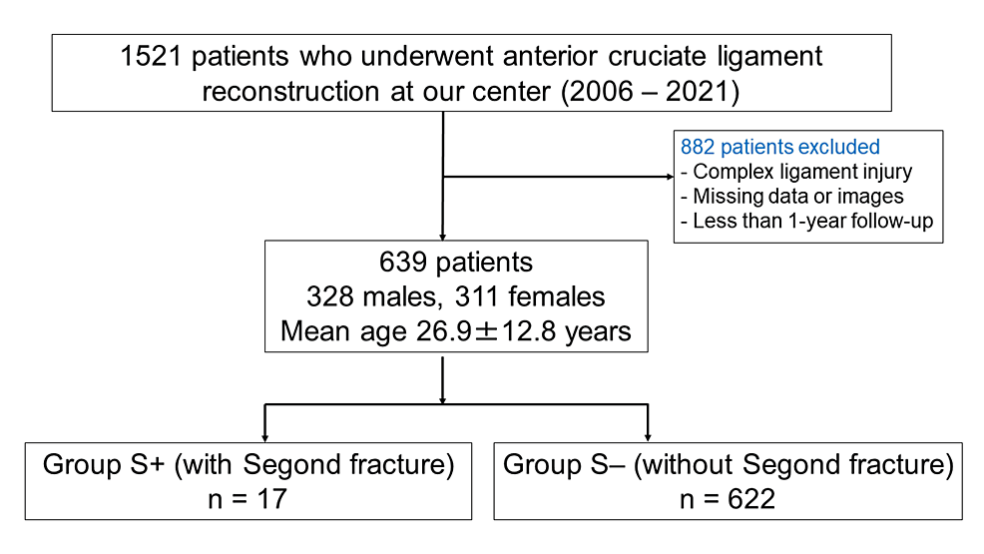
Selection of subjects

A retrospective review of medical records at our center between 2006 and 2021 identified 1521 patients who underwent ACL reconstruction during this period. After excluding patients with postoperative follow-up of less than one year, patients with complex ligament injuries, and patients with missing data or images, 639 patients were included in the analysis. The subjects comprised 328 males and 311 females aged 26.9 ± 12.8 years. They were divided into two groups: those who were diagnosed with Segond fracture based on plain radiographic findings (Group S+, n = 17) and those without Segond fracture (Group S-, n = 622) (Figure 1).

The surgical procedures for ACL reconstruction were reported previously [10]. The second author (SO) conducted or directly supervised all ACL reconstructions, providing guidance for all significant surgical decisions. ACL reconstructions were executed using a single-bundle technique with hamstring tendon autografts. Segond fractures were left unaddressed and not treated in any patient, and ALLs were not subject to reconstruction. Uniform postoperative rehabilitation protocols were adhered to for all patients.

Clinical evaluation was conducted before surgery and 12-18 months (mean 16.2 months) after surgery using the SF-36 as a patient-based assessment method [11], as well as the Lysholm score, visual analog scale (VAS) for knee pain, knee injury and osteoarthritis outcome score (KOOS), and quantitative measurement of knee instability using Telos SE (Telos Japan, Tokyo, Japan).

Patients’ QOL was evaluated with SF-36 before surgery and at the last follow-up after surgery. The results were standardized using the Japanese national standard scores, employing norm-based scoring where absolute scores ranging from 0 to 100 were recalibrated to establish a mean national standard score of 50 with a standard deviation of 10. The SF-36 questionnaire comprises eight subscales, including physical functioning, role-physical, bodily pain, and general health as components of physical health. Additionally, it encompasses vitality, social functioning, role-emotional, and mental health as facets of mental health [11].

Around the same time that the SF-36 survey was conducted, the following evaluations were also performed: Knee pain was assessed using the VAS, which is a clinical pain scale. The knee injury was assessed using the Lysholm knee score scale. The tibial anterior translation was measured on a stress-plain radiograph taken with a Telos SE stress device.

All statistical analyses were performed using StatFlex version 7 (Artec, Osaka, Japan). We utilized mean ± standard deviation for data reporting. A two-group comparison was tested using the Mann-Whitney U test or Fisher's exact test. A p-value of 0.05 or less was deemed statistically significant.

## Results

There were no significant differences in age, percentage of male sex, body mass index, or frequency of sports causing the injury between Group S+ (n = 17) and Group S- (n = 622) (Table 1).

**Table 1:** Demographic and clinical data of study subjects #: Mann-Whitney U test or Fisher's exact test, S+: with Segond fracture, S-: without Segond fracture

	Group S+ (n=17)	Group S- (n=622)	p-value#
Age	25.5 ± 13.6	27.0 ± 12.8	0.30
Male sex, n (%)	12 (70.6)	316 (50.8)	0.14
Body mass index	24.86 ± 3.2	23.6 ± 3.9	0.07
Cause of injury: sports, n (%)	15 (79.6)	495 (88.2)	0.55

**Figure 2:**
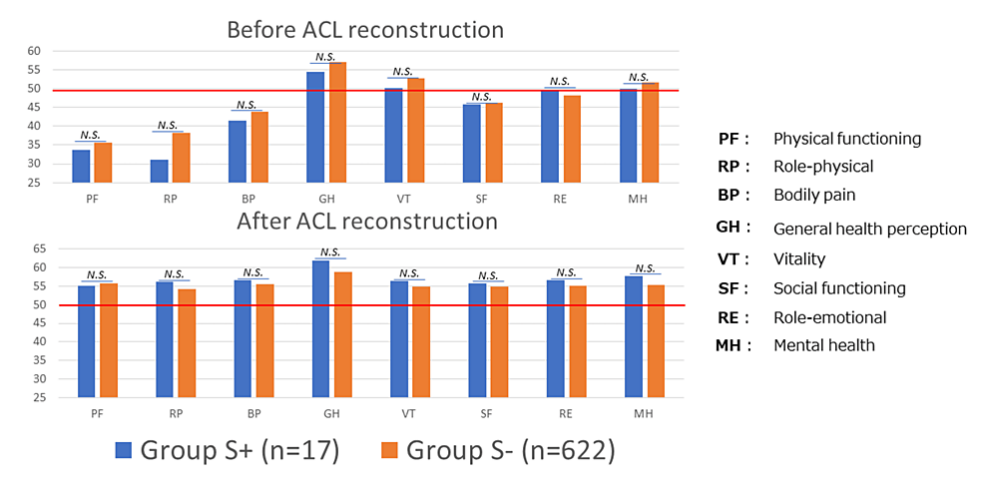
Comparison of subscale scores of SF-36 between Group S+ and Group S- before and after ACL reconstruction The red line at a score of 50 denotes the National standard scores in Japan. N.S.: not significant (Mann-Whitney U test), ACL: anterior cruciate ligament reconstruction, SF-36: 36-item Short Form Survey, Group S+: with Segond fracture, Group S-: without Segond fracture

For the evaluation using SF-36 at the last follow-up, both Group S+ and Group S- improved in all eight subscales compared to before surgery, reaching the national standard scores. No significant differences were noted between the two groups (Figure 2).

At the last follow-up, the mean postop Lysholm score was 93.0 ± 12.1 in Group S+ and 91.7 ± 10.9 in Group S- (P = 0.62); the VAS score for knee pain was 10.0 ± 18.0 in Group S+ and 11.9 ± 16.9 in Group S- (P = 0.62); the total KOOS was 89.0 ± 17.4 in Group S+ and 90.7 ± 9.9 in Group S- (P = 0.39); and the side-to-side difference in tibial anterior translation was 2.8 ± 3.0 mm in Group S+ and 2.7 ± 2.9 mm in Group S- (P = 0.73). No significant differences were observed in all these evaluations (Table 2). No re-rupture occurred in the 17 patients in Group S+ during follow-up.

**Table 2:** Comparison of the results of clinical evaluations between Group S+ and Group S- #: Mann-Whitney U test, *: side-to-side difference in anterior tibial translation, S+: with Segond fracture, S-: without Segond fracture, Preop: before anterior cruciate ligament reconstruction, Postop: after anterior cruciate ligament reconstruction

		Group S+ (n = 17)	Group S- (n = 622)	p-value#
Lysholm	Preop	44.3 ± 16.6	48.4 ± 24.1	0.47
	Postop	93.0 ± 12.1	91.7 ± 10.9	0.62
VAS	Preop	55.2 ± 24.2	50.5 ± 23.7	0.35
	Postop	10.0 ± 18.0	11.9 ± 16.9	0.62
Total KOOS	Preop	45.5 ± 22.1	51.7 ± 23.7	0.24
	Postop	89.0 ± 17.4	90.7 ± 9.9	0.39
Telos*	Preop	3.0 ± 2.2	3.4 ± 3.3	0.88
	Postop	2.8 ± 3.0	2.7 ± 2.9	0.73

## Discussion

Akaki et al. separated the ALS and ACL in stages under arthroscopy in fresh frozen cadaveric knees and quantitatively evaluated the biomechanical function of the ALS using a 3D electromagnetic sensor (EMS) [12]. They reported that the ALS was not involved in the instability during the Lachmann test and pivot shift test. In addition, Miyaji et al. evaluated the presence or absence of anterolateral capsule (ALC) injury using preoperative MRI and compared 42 ALC injury-positive cases with 40 ALC-negative cases with respect to tibial posterior acceleration in the pivot shift test using EMS and other clinical outcomes [13]. They found no differences between the two groups in postoperative tibial posterior acceleration or in postoperative clinical outcomes. Kumahara et al. also studied a cohort of 540 individuals who underwent ACL reconstruction, of whom 22 had concomitant Segond fractures [5]. Their findings revealed that Segond fractures did not affect preoperative or two-year follow-up evaluations of knee stability. Consequently, they concluded that these fractures did not influence the clinical outcomes of primary ACL reconstruction. Therefore, they suggested that treating Segond fractures might not be necessary. Yoon et al. and Melugin et al. also explored the impact of Segond fractures on the outcomes of primary ACL reconstruction [14,15]. Similarly, their findings indicated that the presence of a Segond fracture had no discernible effect on knee laxity in individuals with ACL tears. Furthermore, there were no significant disparities observed in knee stability or clinical scores after ACL reconstruction between patients with and without a Segond fracture. Gaunder et al. conducted a comparison of the occurrence of Segond fractures in patients undergoing primary ACL reconstruction and those undergoing revision reconstruction [16]. They determined that Segond fracture did not emerge as a risk factor for ACL reconstruction failure. In addition, Nagai et al. conducted a meta-analysis on clinical outcomes after ACL reconstruction in patients with concomitant Segond fractures [17]. Their study included five studies with a total of 2418 cases: 304 cases with Segond fracture and 2114 cases without Segond fracture. The rate of revision surgery, IKDC score, Lysholm score, Tegner activity scale, and postoperative knee instability were analyzed. They found no significant differences in clinical outcomes between the presence and absence of Segond fractures and concluded that unrepaired Segond fractures have no significant negative effect on postoperative knee stability or the risk of graft failure or revision surgery after ACL reconstruction.

On the contrary, Sonnery-Cottet et al. proposed the consideration of a combined ACL and ALL reconstruction to enhance rotatory stability control in individuals at elevated risk of graft failure linked to Segond fractures, chronic ACL injuries, and heightened activity levels [18]. Other reports also showed favorable postoperative stability and clinical outcomes after combined ACL and ALL reconstruction [19-23], suggesting that improved rotatory instability and a reduced re-rupture rate may be expected. However, there is a possibility that these studies might have included some cases of non-anatomic ACL reconstruction with additional ALL reconstruction. Future studies should (1) reconfirm anatomic ACL reconstructions and (2) examine strict indications for ALL reconstruction.

In this study, there were no significant differences in postoperative clinical outcomes measured by subjective and objective methods between Group S+ and Group S-, and no re-rupture occurred in the 17 patients in Group S+ during follow-up. Therefore, the presence of a Segond fracture is considered to have little effect on the clinical outcome after ACL reconstruction in patients with concomitant ACL injury and Segond fracture, indicating that treatment of ALS may not be necessary.

The limitations of this study include the infrequent occurrence of Segond fractures among enrolled patients, with the majority not presenting with such fractures. This rarity poses a challenge to achieving a balanced distribution between cases and controls, potentially impacting the statistical power and generalizability of our results. The study's retrospective design, reliance on plain radiographic findings for Segond fracture evaluation, absence of ALS assessment by MRI, and lack of quantitative evaluation of rotatory instability are additional constraints. We acknowledge these limitations and recognize the need for further research to address these challenges and provide more comprehensive insights.

## Conclusions

In our pursuit of elucidating the impact of concomitant Segond fractures with ACL injuries on the outcomes of ACL reconstruction, we conducted a thorough postoperative assessment employing a patient-based and doctor-based evaluation method. The results indicate that the presence of a Segond fracture has minimal influence on the postoperative treatment outcome. Furthermore, our study suggests that the necessity of performing ALS reconstruction is not demonstrated, questioning the routine inclusion of this procedure in the management of ACL injuries.

Our findings contribute to the growing body of evidence suggesting that Segond fractures play a limited role in influencing the outcomes of ACL reconstruction. The observed minimal impact on postoperative treatment outcomes raises important considerations regarding the routine incorporation of ALS reconstruction, highlighting the need for further research to refine treatment strategies and better delineate the indications for specific interventions in the context of ACL injuries associated with Segond fractures.
